# High Level of Legumain Was Correlated With Worse Prognosis and Peritoneal Metastasis in Gastric Cancer Patients

**DOI:** 10.3389/fonc.2020.00966

**Published:** 2020-07-16

**Authors:** Yan Wang, Shilong Zhang, Haiwei Wang, Yuehong Cui, Zhiming Wang, Xi Cheng, Wei Li, Jun Hou, Yuan Ji, Tianshu Liu

**Affiliations:** ^1^Department of Medical Oncology, Zhongshan Hospital, Fudan University, Shanghai, China; ^2^Minhang Hospital, Fudan University, Shanghai, China; ^3^Maternity and Children's Hospital of Fujian Province, Fujian Medical University, Fuzhou, China; ^4^Department of Pathology, Zhongshan Hospital, Fudan University, Shanghai, China; ^5^Center of Evidence-Based Medicine, Fudan University, Shanghai, China

**Keywords:** gastric cancer, peritoneal metastasis, legumain, survival, nomogram

## Abstract

**Background:** Accumulating evidence has demonstrated that legumain (LGMN) is abnormally expressed in several malignancies and functions as an oncogene. However, the association between LGMN and gastric cancer (GC) has not yet been fully elucidated. In this study, we performed a comprehensive analysis of the role of LGMN in clinicopathologic characteristics and survival of GC patients.

**Methods:** The study had two patient cohorts, The Cancer Genome Atlas (TCGA) cohort and the Zhongshan Hospital cohort, both of which were used to analyze the role of LGMN in GC samples. The relationship between LGMN and clinicopathologic characteristics was determined by the Chi-square test and logistic regression analysis. The Kaplan–Meier method and Cox proportional hazards regression analysis were conducted to investigate the prognostic role of LGMN in GC patients. Moreover, a nomogram was constructed based on the factors that were independently associated with peritoneal metastasis. Finally, the gene set enrichment analysis (GSEA) was conducted to explore the underlying pathways through which LGMN was involved in GC progression.

**Results:** The mRNA and protein levels of LGMN were significantly upregulated in GC tissues, especially for diffuse-type GC. High level of LGMN was independently associated with poor prognosis in both TCGA and Zhongshan cohorts. Further analysis showed that increased protein level of LGMN was related to peritoneal metastasis in GC patients. In a nomogram model, the LGMN expression could help predict the possibility of peritoneal metastasis in GC patients. LGMN was a strong determinant for prediction of peritoneal metastasis. GC patients with high LGMN expression tended to have worse survival together with more frequent diffuse-type tumors and increased risk of peritoneal metastasis. The GSEA results showed that focal adhesion, ecm receptor interaction, cell adhesion molecules cams, TGF-β signaling pathway, JAK-STAT signaling pathway, gap junction, etc. were differentially enriched in the phenotype with high LGMN expression.

**Conclusion:** LGMN was an independent prognostic factor for OS in GC patients. Increased expression of LGMN was significantly associated with peritoneal metastasis. The nomogram based on LGMN might guide the clinical decisions for patients with GC.

## Introduction

Gastric cancer (GC) is one of the common malignant tumors threatening human health, causing ~1,033,701 new cases and 782,685 deaths worldwide in 2018 (1). According to Lauren's classification system, GC has three types, intestinal type, diffuse type, and mixed type, of which the diffuse type tends to be more invasive. Peritoneal metastasis accounts to nearly 50% of death in GC patients (2, 3). Interestingly, peritoneal metastasis is more commonly observed in diffuse-type GC than other types (4–6), which may contribute to their worse survival. Although considerable advances have been made in the management of GC, such as chemotherapy, targeted therapy, and immunotherapy, the 5-year overall survival (OS) of GC patients with peritoneal metastasis remains dismal (7, 8). However, the molecular biomarkers and mechanisms underlying peritoneal metastasis have not been well-established in GC patients. Therefore, it is essential to identify novel molecular biomarkers for early diagnosis, prevention, and targeted therapy for GC patients.

Legumain (LGMN), also known as asparagine endopeptidase, is a cysteine endopeptidase of the asparaginyl endopeptidase family, showing high specificity for hydrolysis of asparaginyl bonds (9). It belongs to the peptidase family C13, which expresses both on surface and intracellularly (10). LGMN promotes activation of zymogen gelatinase A through cleaving pro-gelatinase A, which is considered to play a critical role in extracellular matrix degradation and remodeling, thereby facilitating cell migration and invasion (11–13). Our recent study has demonstrated that LGMN is expressed at elevated levels in diffuse GC cell lines and contributes critically to the invasion and metastasis phenotype through epithelial–mesenchymal transition in diffuse GC (14). Previous studies have shown that higher LGMN level is associated with poor prognosis of multiple cancers including breast cancer (15), colorectal cancer (16), and prostate cancer (17). However, the exact relationship of LGMN expression and clinicopathologic signature, especially peritoneal metastasis, in GC patients remains poorly characterized. To our best knowledge, there is no literature reporting on a clinicopathologic signature to improve the diagnosis and prediction of peritoneal metastasis in GC patients.

Therefore, this study aimed to investigate the expression pattern of LGMN in GC tissue from the Zhongshan hospital cohort and to use bioinformatics data from The Cancer Genome Atlas (TCGA) to explore the role of LGMN as a clinicopathological and prognostic biomarker for patients with GC. Moreover, the nomogram integrating LGMN expression and clinical clinicopathologic characteristics was also established to predict peritoneal metastasis for GC patients.

## Materials and Methods

### Extraction of Clinical and mRNA Expression Data From TCGA Cohort

The mRNAs expression data and corresponding clinicopathologic information of GC patients were downloaded from the TCGA database (up to January 1, 2019). The included clinical characteristics were age, gender, pathologic grade, tumor stage, survival time, and vital status. Patients were excluded if they had incomplete survival information or their survival time was 0 days. The baseline characteristics of GC patients in the TCGA cohort are summarized in Supplement Table 1.

### Patients in the Zhongshan Hospital

A total of 139 patients who were diagnosed with advanced GC at the Department of Medical Oncology, Zhongshan Hospital, Fudan University, Shanghai, China, from January 2009 and June 2016 were included in our analysis. Inclusion criteria for the eligible patients were listed as follows: (a) histologically proven gastric adenocarcinoma; (b) no previous anticancer treatment; (c) signs of distant metastasis; (d) completed clinicopathological and follow-up information. Written informed consent from all patients was obtained with the approval of the Ethics Committee of Zhongshan Hospital. The primary outcome is OS, which was censored at the last follow-up record (December 31, 2017). The baseline characteristics of GC patients in the Zhongshan cohort are summarized in Supplement Table 2.

### IHC Staining and Evaluation of IHC Intensity

Immunohistochemistry was performed on tissue microarray (TMA) according to the standard biotin–streptavidin–peroxidase method (18). The polyclonal goat anti-human LGMN antibody (#AF2199, R & D Systems, USA) in a 1:300 dilution was used for IHC staining. The IHC results were analyzed by two independent pathologists who were blinded to the clinical characteristics. Staining intensity for LGMN was scored as 0 (0%), 1 (<10%), 2 (10–50%), and 3 (>50%), depending on the percentage of positive-stained cells. In subsequent statistical analysis, specimens with a score of ≤2 were grouped as low LGMN expression, while a score of 3 was grouped as high LGMN expression. The specimens would be reexamined by both pathologists under a multihead microscope in case of a discrepancy in scoring.

### Western Blot

The GC cell lines were maintained in RPMI 1640 containing 10% FB. Cellular protein was extracted using a protein extraction kit, according to the manufacturer's instructions (#WLA019, Wanleibio, China). Proteins were separated using 6% SDS-PAGE gel electrophoresis and then transferred to PVDF membranes. The membranes were blocked in 5% non-fat dry milk in Tris-buffered saline (pH 7.5) for an hour at 37°C. Membranes were incubated overnight at 4°C with anti-human LGMN antibody as IHC described above, then followed by the horseradish peroxidase conjugated secondary antibody for 1 h at room temperature. Signals were detected using enhanced chemiluminescence reagents (Pierce, Rockford, IL, USA).

### GSEA Enrichment

The gene set enrichment analysis (GSEA) created a list of all genes connected with the expression of the LGMN. Then, the samples were categorized as the high- and low-LGMN phenotypes to elucidate the potential biological function utilizing GSEA software GSEA v2.2.2 (19). The annotated gene sets c2.cp.kegg.v7.0.symbols.gmt in the MSigDB Collection were utilized as the reference gene sets. The nominal *P*-value and normalized enrichment score (NES) were used to sort the pathways enriched in each phenotype. Gene sets with nominal *P* < 0.05 and FDR <0.25 were considered statistically significant.

### Statistical Analysis

The relationship between LGMN expression and clinicopathological characteristics was analyzed with Chi-square test and logistic regression. The Kaplan–Meier method and log-rank test were used to perform survival analysis. Univariate and multivariate Cox proportional hazards regression analysis were used to evaluate whether LGMN could be an independent prognostic factor in GC. We used the “rms” R package to plot the nomogram for peritoneal metastasis prediction among GC patients. Receiver operating characteristics (ROC) curve was used to evaluate the performance of nomogram in peritoneal metastasis prediction among GC patients. Decision curve analysis (DCA) was introduced to assess the clinical utility of this nomogram (20). DCA is a novel analytical technique that integrates all clinical consequences of a decision and then quantifies the clinical utility of a predictive model (21). All analyses were conducted using R software (version 3.5.1). *P* < 0.05 was considered to be statistically significant.

## Results

### The Level of LGMN Was Upregulated in GC, Especially for Diffuse-Type GC

**Figure 1:**
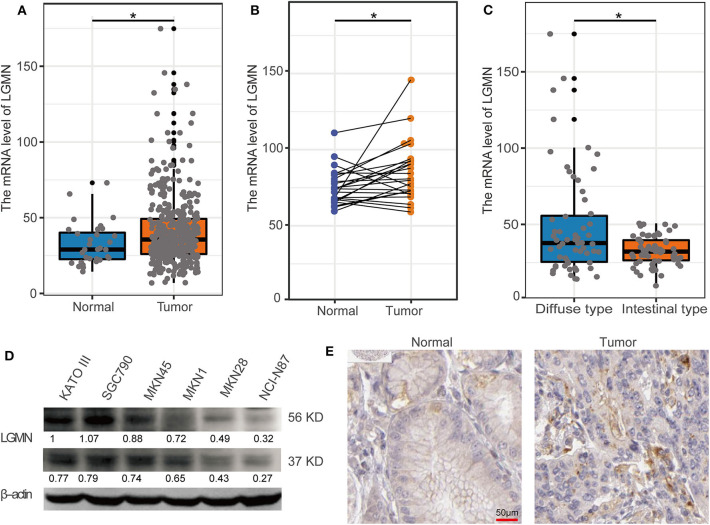
The level of LGMN in GG based on TCGA database, Western blot, and HPA database. **(A)** LGMN expression level in GC tissues relative to corresponding normal gastric tissue from the TCGA database. **(B)** Comparison of LGMN expression in 24 matched GC tissues and normal tissues. **(C)** Comparison of LGMN expression between diffuse-type GC and intestinal-type GC from the TCGA database. **(D)** Comparison of LGMN expression between diffuse-type GC and intestinal-type GC in different cell lines by Western blot. **(E)** Representative images of protein expression detected by immunohistochemistry of LGMN were detected in GC and normal tissues from the HPA database. *P < 0.05.

First, the TCGA database was used to examine the differential expression levels of LGMN mRNA between GC and normal gastric tissue. The LGMN mRNA expression level was significantly higher in GC tissues than in normal tissues (*P* < 0.05, Figure 1A). Additionally, paired analysis of LGMN mRNA expression in 24 matched GC tissues and normal tissues demonstrated that LGMN mRNA expression was significantly increased in tumor tissues compared with normal tissues (*P* < 0.05, Figure 1B). Interestingly, we found that the mRNA levels of LGMN were higher in diffuse-type GC compared with intestinal-type GC (*P* < 0.05, Figure 1C). To further confirm this result, we performed Western blot to compare the LGMN expression in three cell lines of diffuse-type GC (KATO III, SGC790, and MKN45) between three cell lines of intestinal-type GC (MKN1, MKN28, and NCI-N87). The Western blot results demonstrated that diffuse-type cells showed a higher expression of LGMN compared with the intestinal-type GC (Figure 1D). Additionally, representative images from the Human Protein Atlas (HPA) database demonstrated that LGMN protein expression was higher in GC tissues compared with normal gastric tissues (Figure 1E).

### LGMN Was an Independently Prognostic Factor in GC Patients

**Figure 2:**
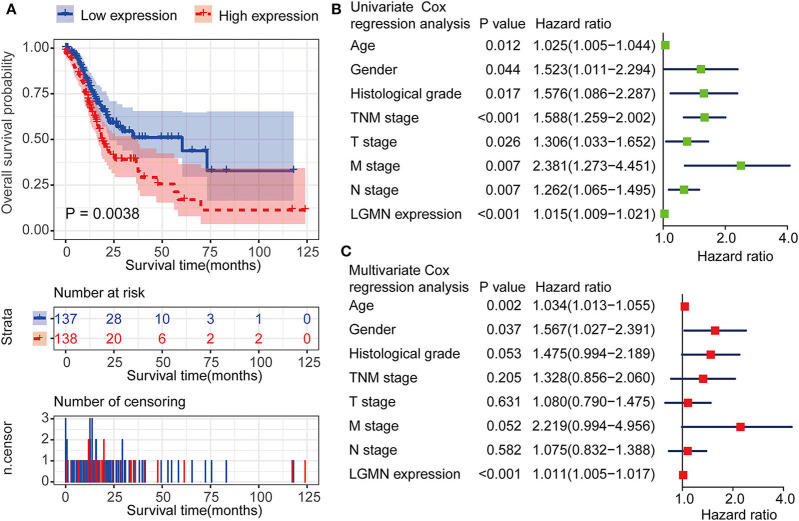
LGMN expression was an independent prognostic factor associated with OS in the GC patients from the TCGA cohort. **(A)** Kaplan–Meier survival analysis between GC patients in the high- and low-expression group of LGMN. **(B)** Univariate Cox proportional hazards regression analyses of overall survival in GC patients. The green squares on the transverse lines represent the HR, and the blue transverse lines represent 95% CI. **(C)** Multivariate Cox proportional hazards regression analyses of overall survival in GC patients. The red squares on the transverse lines represent the HR, and the blue transverse lines represent 95% CI.

In the TCGA database, GC patients were divided into the high-expression group and the low-expression group using median value as a cutoff (35.62). The Kaplan–Meier analysis showed that the GC patients with high mRNA level of LGMN had an unfavorable OS, and the median OS for the high LGMN group and the low LGMN group was 18.47, and 34.77 months, respectively (*P* = 0.0038) (Figure 2A). In the Cox proportional hazards regression analysis, we discovered that GC patients with high mRNA level of LGMN or high histological grade (G3/4) were at significantly high risk of death. GC patients with a higher age or distant metastasis were also at high risk of death (Figure 2B). After adjustment for age, gender, tumor stage, and histological grade, to our surprise, high mRNA level of LGMN remained associated with high risk of death in GC patients (HR, 1.011; 95% CI, 1.005–1.017; *P* < 0.001, Figure 2C).

**Figure 3:**
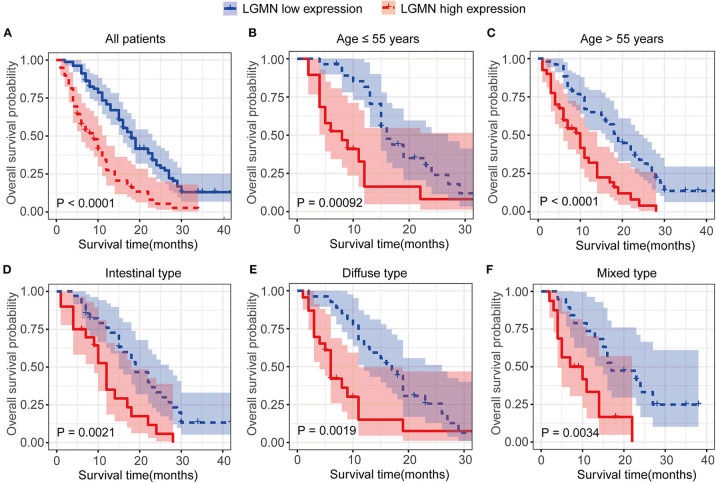
Kaplan–Meier survival analyses of GC patients from the TCGA cohort. **(A)** The Kaplan–Meier curves for all patients set. **(B)** The Kaplan–Meier curves for age ≥ 55 years subgroup. **(C)** The Kaplan–Meier curves for age < 55 years subgroup. **(D)** The Kaplan–Meier curves for the intestinal-type subgroup. **(E)** The Kaplan–Meier curves for the diffuse-type subgroup. **(F)** The Kaplan–Meier curves for the mixed-type subgroup.

We next ask whether the prognostic value of LGMN persisted in the protein level. TMA derived from 139 GC patients in the Zhongshan cohort was used. In univariate Cox proportional hazards regression analysis, GC patients with high LGMN expression had a significantly lower 1-year OS than those with low LGMN expression (27.54 vs. 70. 90%, *P* < 0.0001) (Figure 3A, Table 1). In addition, tumor site (*P* = 0.027) and recurrence (*P* = 0.003) were also significantly associated with OS. Multivariate Cox proportional hazards regression analysis was performed using all of the significant variables in the univariate analysis. The results from the multivariate analysis showed that LGMN expression was a significantly independent prognostic factor for OS (*P* < 0.001). Of note, high expression level of LGMN might double the risk of death among GC patients (HR, 2.51; 95% CI, 1.68–3.76; *P* < 0.001) (Table 1). We further conducted a subgroup analysis for evaluating the effect of LGMN expression on OS based on two risk factors, namely, age and Lauren type. We found that high expression of LGMN continued to contribute to a worse survival even in each subgroup stratified by age (Figures 3B,C) and Lauren type (Figures 3D–F).

**Table 1:** Univariate and multivariate Cox proportional hazards regression analysis of the overall survival in GC patients from the Zhongshan cohort.

**Variables**	**Overall survival**			
	**Univariate**	***P-*value**	**Multivariate**	***P-*value**
	**analysis**		**analysis**	
**Age**				
≤55	Reference		/	
>55	1.18 (0.81–1.71)	0.379	/	/
**Gender**				
Male	Reference		/	
Female	1.04 (0.71–1.53)	0.841	/	/
**Tumor site**				
Cardia	Reference		Reference	
Corpus	1.46 (0.84–2.44)	0.184	1.21 (0.71–2.07)	0.481
Antrum	3.48 (1.15–10.52)	0.027	0.91 (0.29–2.83)	0.874
**Lauren type**				
Intestinal type	Reference		/	/
Diffuse type	1.39 (0.90–2.12)	0.134	/	/
Mixed type	1.06 (0.65–1.72)	0.812	/	/
**Historical grade**				
G1/G2	Reference		/	/
G3/G4	1.21 (0.78–1.86)	0.397	/	/
**Her2 status**				
Negative	Reference		/	
Positive	1.03 (0.61–1.73)	0.918	/	/
**Tumor recurrence**				
No	Reference		Reference	
Yes	1.78 (1.21–2.61)	0.003	0.68 (0.45–1.02)	0.059
**LGMN expression**				
Low	Reference		Reference	
High	2.78 (1.89–4.09)	<0.001	2.51 (1.68–3.76)	<0.001

### Increased Protein Level of LGMN Was Related to Peritoneal Metastasis in GC Patients

**Figure 4:**
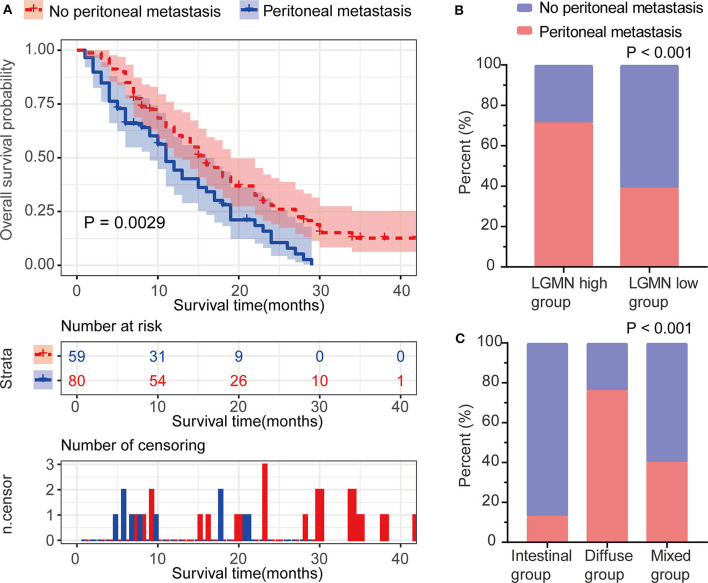
The association between LGMN and peritoneal metastasis in GC patients from the Zhongshan cohort. **(A)** The Kaplan–Meier survival analyses of peritoneal metastasis in GC patients. **(B)** The percentage of peritoneal metastasis in high/low LGMN level of GC tissues was compared. **(C)** The Lauren type in GC patients with and without peritoneal metastasis was compared.

Peritoneal metastasis is one of the most common causes of death in GC patients. In the Zhongshan cohort, we observed that patients with peritoneal metastasis had a significantly increased risk of death in GC (Figure 4A). Meanwhile, using the median expression score as the cutoff point, we tested the probability of peritoneal metastasis in the low LGMN and high LGMN expression groups using Chi-square test (Table 2). In total, 71.25% patients with high LGMN expression had peritoneal metastasis, but only 38.98% patients with low LGMN expression had metastasis (Chi-square test, *P* < 0.001; Figure 4B). At the same time, patients with diffuse-type GC tended to suffer from peritoneal metastasis compared to those patients with intestinal GC and those with mixed GC (*P* < 0.001; Figure 4C). These results were further confirmed by logistic regression analysis (Table 3). Additionally, we found that female patients were more likely to progress to peritoneal metastasis (OR = 4.633; 95% CI, 1.835–12.449; *P* = 0.001).

**Table 2:** Chi-square tests for patients stratified by peritoneal metastasis status from the Zhongshan cohort.

**Variables**	**Peritoneal metastasis status**		***P*-value**
	**Metastasis (%)**	**Without metastasis (%)**	
	**59(42.5)**	**80 (57.5)**	
**Gender**			<0.001
Male	26 (44.1)	61 (76.2)	
Female	33 (55.9)	19 (23.8)	
**Age**			0.099
≤55	25 (42.4)	22 (27.5)	
>55	34 (57.6)	58 (72.5)	
**Tumor site**			0.004
Cardia	4 (6.8)	19 (23.8)	
Corpus	55 (93.2)	57 (71.2)	
Antrum	0 (0.0)	4 (5.0)	
**Lauren type**			<0.001
Intestinal type	7 (11.90)	47 (58.8)	
Diffuse type	38 (64.4)	12 (15.0)	
Mixed type	14 (23.7)	21 (26.2)	
**LGMN expression**			<0.001
High	36 (61.0)	23 (28.7)	
Low	23 (39.0)	57 (71.3)	
**Histological grade**			0.096
G1/G2	9 (15.3)	23 (28.8)	
G3/G4	50 (84.7)	57 (71.2)	
**Her2 status**			0.109
Positive	4 (6.8)	14 (17.5)	
Negative	55 (93.2)	66 (82.5)	
**Tumor recurrence**			0.083
Yes	29 (49.2)	48 (60.0)	
No	30 (50.8)	32 (40.0)	
**Surgery**			0.669
Done	34 (57.6)	42 (52.5)	
Not done	25 (42.4)	38 (47.5)	
**Chemotherapy**			0.102
Done	54 (91.5)	64 (80.0)	
Not done	5 (8.5)	16 (20.0)	

**Table 3:** LGMN expression associated with peritoneal metastasis in GC patients from the Zhongshan cohort.

**Variables**	**Logistic regression**		
	**OR in peritoneal metastasis**	**95% CI of OR**	***P*-value**
**Age** (≤55 vs. >55)	0.771	0.294–2.029	0.596
**Gender** (male vs. female)	4.633	1.835–12.449	0.001
**Tumor site** (cardia vs. corpus)	1.558	0.421–6.328	0.514
(Cardia vs. antrum)	10.584	0.764–152.882	0.071
**Lauren type** (intestinal vs. diffuse type)	19.461	5.312– 87.653	<0.001
(Intestinal vs. mixed type)	2.736	0.808–9.771	0.109
**Histological grade** (G1/G2 vs. G3/G4)	0.916	0.221–4.198	0.889
**Her2 status** (negative vs. positive)	0.533	0.107–2.846	0.443
**LGMN expression** (low vs. high)	3.941	1.558–10.770	0.005
**Tumor recurrence**(no vs. yes)	2.046	0.831–5.197	0.123

**Figure 5:**
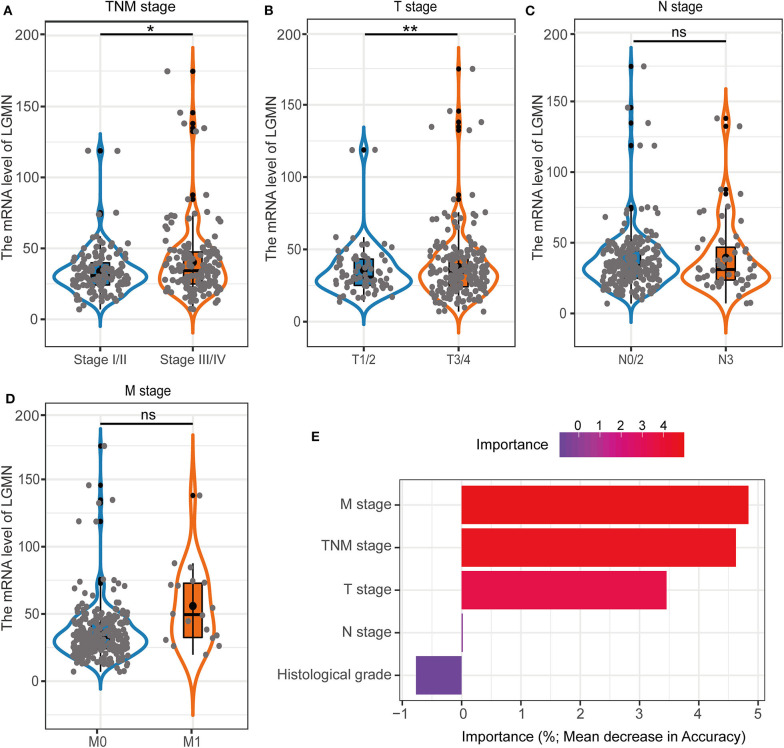
The association with LGMN expression and clinicopathologic characteristics including **(A)** TNM stage, **(B)** M stage, **(C)** T stage, and **(D)** N stage in the TCGA cohort. **(E)** M stage contributed most to classification between high LGMN and low LGMN patients by RandomForest in the TCGA cohort. *P < 0.05, **P < 0.01, ns, no significance.

In the TCGA cohort, we first investigated the LGMN mRNA levels in different tumor stages. We found that the LGMN expression was much higher (*P* < 0.05) in GC patients with stage III/IV compared to GC patients with tumor stage I/II (Figure 5A). Interestingly, similar results were obtained in the M stage, as LGMN expression was associated with high M stage (Figure 5B). However, with the increased T or N stage, the LGMN expression was not further increased (Figures 5C,D). These results indicated that high expression of LGMN might contribute to advanced tumor stage mainly through promoting distant metastasis. Furthermore, we performed unsupervised RandomForest classification analysis to validate our result, which determined that the M stage contributes most to discrimination between high LGMN and low LGMN samples (Figure 5E). Since the TCGA database did not record the peritoneal metastasis status for GC patients, we failed to evaluate the role of LGMN mRNA played in the peritoneal metastasis. However, we found that the LGMN mRNA expression was much higher in diffuse GC patients compared to intestinal ones (Figure 1C), consistent with the observations in the Zhongshan cohort.

### The Protein Level of LGMN, Combined With Lauren Type and Gender, Was Able to Better Predict Peritoneal Metastasis for GC Patients

**Figure 6:**
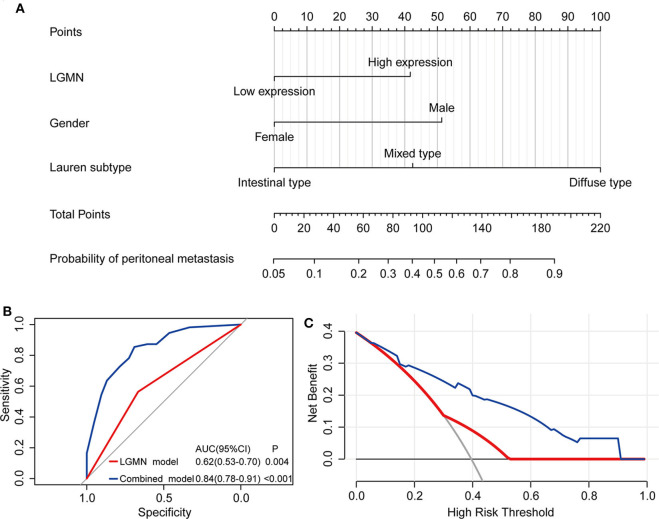
The role of LGMN in predicting peritoneal metastasis in GC patients from the Zhongshan cohort. **(A)** Nomograms for predicting peritoneal metastasis. **(B)** ROC analyses of the nomogram for peritoneal metastasis prediction. **(C)** DCA for assessment of the clinical utility of the nomogram.

The above results indicated that the level of LGMN, Lauren type, and gender might be related to peritoneal metastasis in GC patients. Therefore, a nomogram for prediction of peritoneal metastasis probabilities, which included LGMN, Lauren type, and gender were constructed (Figure 6A). ROC curve was used to analyze the power of LGMN and nomogram to discriminate between GC patients with or without peritoneal metastasis. According to the ROC analysis, the area under the curve (AUC) of the nomograms for probability based on LGMN and nomogram (Figure 6B) was 0.615 and 0.842, respectively, suggesting that this model can accurately predict the possibility of potential peritoneal metastasis among GC patients. After addressing the accuracy, DCA was introduced to evaluate the clinical utility of this nomogram. Figure 6C showed that the established nomogram had high potential for clinical application.

### The Potential Molecular Mechanisms Mediated by LGMN in GC

**Figure 7:**
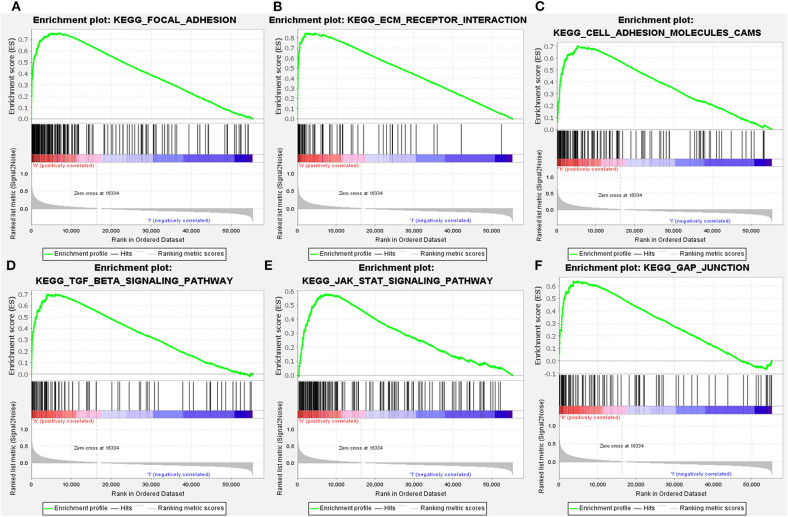
GSEA analyses of KEGG signaling pathways activated in GC patients with high expression of LGMN compared with the ones with low expression. **(A)** Focal adhesion, **(B)** ecm receptor interaction, **(C)** cell adhesion molecules cams, **(D)** TGF-β signaling pathway, **(E)** JAK-STAT signaling pathway, and **(F)** gap junction were differentially enriched when LGMN overexpressed.

Since LGMN was upregulated and an independent prognostic factor was associated with OS in both cohorts, we were eager to explore the underlying mechanisms by which LGMN is involved in GC progression. Next, GSEA was performed between patients with low or high LGMN mRNA expression based on the TCGA cohort. Based on the NESs, the several significantly enriched signaling pathways were selected (Figures 7A–F). The focal adhesion, ecm receptor interaction, cell adhesion molecules cams, TGF-β signaling pathway, JAK-STAT signaling pathway, gap junction, etc. were differentially enriched in phenotypes with high LGMN expression. The top 20 enriched signaling pathways were summarized in Table 4. In conclusion, functional enrichment analysis results showed that LGMN might play a significant role in GC progression and biological progress.

**Table 4:** Gene sets enriched in the high-expression phenotype of GC patients from the TCGA cohort.

**Name**	**ES**	**NES**	**NOM**	**FDR**
			***P*-value**	***Q*-value**
KEGG_FOCAL_ADHESION	0.76	2.57	0.00	0.00
KEGG_ECM_RECEPTOR_INTERACTION	0.85	2.50	0.00	0.00
KEGG_DILATED_CARDIOMYOPATHY	0.72	2.39	0.00	0.00
KEGG_HYPERTROPHIC_CARDIOMYOPATHY_	0.71	2.39	0.00	0.00
HCM				
KEGG_CYTOKINE_CYTOKINE_RECEPTOR_	0.64	2.39	0.00	0.00
INTERACTION				
KEGG_HEDGEHOG_SIGNALING_PATHWAY	0.73	2.34	0.00	0.00
KEGG_PATHWAYS_IN_CANCER	0.63	2.33	0.00	0.00
KEGG_TGF_BETA_SIGNALING_PATHWAY	0.70	2.32	0.00	0.00
KEGG_REGULATION_OF_ACTIN_	0.61	2.32	0.00	8.32E−05
CYTOSKELETON				
KEGG_AXON_GUIDANCE	0.64	2.27	0.00	1.46E−04
KEGG_GAP_JUNCTION	0.64	2.26	0.00	1.33E−04
KEGG_CELL_ADHESION_MOLECULES_	0.70	2.25	0.00	1.67E−04
CAMS				
KEGG_BASAL_CELL_CARCINOMA	0.71	2.25	0.00	1.55E−04
KEGG_MELANOMA	0.64	2.23	0.00	1.45E−04
KEGG_HEMATOPOIETIC_CELL_LINEAGE	0.71	2.23	0.00	1.36E−04
KEGG_CALCIUM_SIGNALING_PATHWAY	0.58	2.20	0.00	2.16E−04
KEGG_GLYCOSAMINOGLYCAN_	0.77	2.19	0.00	4.45E−04
DEGRADATION				
KEGG_MAPK_SIGNALING_PATHWAY	0.57	2.19	0.00	4.21E−04
KEGG_JAK_STAT_SIGNALING_PATHWAY	0.58	2.16	0.00	5.42E−04
KEGG_RENAL_CELL_CARCINOMA	0.65	2.15	0.00	6.43E−04

*NES, normalized enrichment score; NOM, nominal; FDR, false discovery rate*.

## Discussion

Although LGMN has been confirmed to be highly expressed in several types of solid tumors (15–17), its expression level and potential clinical implications in GC, which were the focus of the current study, have not been well-defined. This study represented the first comprehensive and detailed analysis of LGMN in GC patients from the TCGA database and our institute to investigate its association with clinicopathologic characteristics, survival, function, and expression difference. By analyzing GC patients from the TCGA cohort and the Zhongshan cohort, we demonstrated a notable association between high LGMN expression and poor survival in GC patients. Moreover, LGMN expression has also been demonstrated as an independent prognostic factor for OS, and higher LGMN levels in patients with peritoneal metastasis and diffuse-type GC were observed, which suggested that LGMN might play a vital role in the peritoneal metastasis of GC. Furthermore, LGMN could be integrated with acknowledged clinicopathological factors to construct a nomogram for peritoneal metastasis prediction.

Our recent study has demonstrated that LGMN is highly expressed in diffuse-type GC cell lines and enhances the malignant phenotype of diffuse-type GC, including proliferation, invasion, as well as metastasis (14). However, its clinical implications for GC patients have not been investigated. Additionally, although Li et al. have reported the relationship of overexpression of LGMN and poor prognosis of GC (22), the exact correlation of LGMN and peritoneal metastasis in GC is still unknown. Peritoneal metastasis, as the most critical determinant of death in GC patients (2), is difficult to discriminate from advanced GC preoperatively (23). In most cases, peritoneal metastasis may remain asymptomatic for a remarkably long period of time and therefore is typically diagnosed intraoperatively, which does not benefit surgeons in determining the optimal therapeutic strategy (23). Operative diagnostic methods such as staging microscopy have emerged as a standard method for discrimination of peritoneal metastasis among GC patients (24, 25). Nevertheless, these methods have an invasive nature, are time-consuming, are expensive, and result in complications including intra-abdominal organ iatrogenic damages, hemorrhage, as well as infections (26). Recently, the main non-invasive diagnostic methods for peritoneal metastasis are imaging examinations, such as computed tomography (CT), positron emission tomography–computed tomography (PET-CT), and magnetic resonance imaging (MRI); however, all of them lack diagnostic accuracy for early micrometastatic lesions (27, 28).

In recent years, researches had undertaken efforts to develop several biomarkers in identifying GC patients with peritoneal metastasis (29–31). However, most of them mainly focus on the clinicopathological parameters and ignore the components of genetic characteristics, which also play a critical role in peritoneal metastasis (32). It is reasonable to combine clinicopathological parameters and gene expression for better prediction and clinical application. In the Zhongshan cohort, we tested the probability of peritoneal metastasis between GC patients with low and high LGMN expression. We found that patients with high LGMN expression had increased risks of peritoneal metastasis compared to those with low LGMN expression. The poor prognosis of patients with high LGMN expression might derive from higher rate of peritoneal metastasis. Hence, a nomogram was constructed by integrating Lauren type, gender, and LGMN expression. Notably, this nomogram indicated that LGMN was a strong determinant for peritoneal metastasis prediction. In addition, the nomogram showed satisfactory performance, as indicated by ROC curves and DCA. The nomogram might be useful for patient counseling and individualized clinical decision-making as it helps predict the possibility that GC patients will encounter peritoneal metastasis.

There are also several limitations about our present study. First, as a retrospective study, it has several inherent limitations, such as selection bias confounding factors and missing data, which might provide inaccurate conclusions (33). Therefore, to further confirm our results, a prospective study with large samples might be needed. Second, the Zhongshan cohort consisted of GC patients who undertook previous surgery; hence, the limited sample size might weaken the power of LGMN as a biomarker for detecting peritoneal metastasis. In addition, as we used the TCGA cohort as well as a clinical cohort for analysis, the clinicopathological factors and expression profiles were different between cohorts. Third, although the biologic effect including invasion and migration has been demonstrated in our recent publication (14), this study failed to explore the underlying mechanisms of the signaling pathways involved in GC, but a GSEA was performed. Further studies are required to investigate the mechanisms responsible for the regulation of LGMN and its role in peritoneal metastasis in GC, which would provide insights into its roles in other malignancies. Nevertheless, we have provided strong evidence indicating that LGMN is overexpressed in GC and is associated with a poor survival for GC patients. What is more, our data suggested that LGMN might be of a critical role in the progression of peritoneal metastasis and could be integrated with the acknowledged clinicopathological factors to predict the possibility of peritoneal metastasis, which might guide the clinical management.

In conclusion, to the best of our knowledge, this is the first comprehensive analysis of expression pattern and clinicopathological implications of LGMN in GC. This study demonstrated that higher levels of LGMN mRNA and protein were observed in GC compared to their adjacent tissues. LGMN expression was an independent prognostic factor associated with OS. Moreover, higher LGMN levels tended to be observed patients with diffuse-type GC and peritoneal metastasis. Furthermore, a nomogram for peritoneal metastasis prediction was constructed by Lauren type, gender, and LGMN expression, which show satisfactory performance and clinical utility, which might guide patient counseling and clinical decision-making.

## Data Availability Statement

Publicly available datasets were analyzed in this study, these can be found in the Cancer Genome Atlas (https://portal.gdc.cancer.gov/). The raw data supporting the conclusions of this article will be made available by the authors, without undue reservation, to any qualified researcher.

## Ethics Statement

The studies involving human participants were reviewed and approved by the Ethics Committee of Zhongshan Hospital, Fudan University. The patients/participants provided their written informed consent to participate in this study.

## Author Contributions

YW and TL contributed to the conception, design, and drafting of the manuscript. YC, ZW, XC, and WL obtained ethical approval and contributed to the preparation of the dataset. YW, SZ, and HW carried out the statistical analysis. TL, YJ, YW, and SZ contributed with a critical revision of the manuscript. All authors contributed to the article and approved the submitted version.

## Conflict of Interest

The authors declare that the research was conducted in the absence of any commercial or financial relationships that could be construed as a potential conflict of interest.
