# Supplementary material for: High Level of Legumain Was Correlated With Worse Prognosis and Peritoneal Metastasis in Gastric Cancer Patients
Source: Front Oncol. 2020 Jul 16;10:966. doi: 10.3389/fonc.2020.00966 (PMC7378441; doi:10.3389/fonc.2020.00966)
Supplement: Supplementary file 1 [file Table_1.DOCX]

**Supplement table 1.** Baseline Characteristics of GC patients in the TCGA Cohort (N=443).

| Clinical features | n | % |
| --- | --- | --- |
| Gender |  |  |
| Male | 285 | 64.3 |
| Female | 158 | 35.7 |
| Lauren type |  |  |
| Intestinal type | 72 | 16.2 |
| Diffuse type | 191 | 43.3 |
| Mixed type or unknow | 180 | 40.5 |
| T stage |  |  |
| T1 | 23 | 5.2 |
| T2 | 93 | 21.0 |
| T3 | 198 | 44.7 |
| T4 | 119 | 26.8 |
| Unknow | 10 | 2.3 |
| N stage |  |  |
| N0 | 132 | 29.8 |
| N1 | 119 | 26.8 |
| N2  N3 | 86  88 | 19.4  19.8 |
| Unknow | 18 | 4.2 |
| M stage |  |  |
| M0 | 397 | 89.6 |
| M1 | 46 | 10.4 |
| TNM |  |  |
| I | 59 | 13.3 |
| II | 131 | 29.6 |
| III | 187 | 42.2 |
| IV | 46 | 10.4 |
| Unknow | 20 | 4.5 |
