# Supplementary material for: High Level of Legumain Was Correlated With Worse Prognosis and Peritoneal Metastasis in Gastric Cancer Patients
Source: Front Oncol. 2020 Jul 16;10:966. doi: 10.3389/fonc.2020.00966 (PMC7378441; doi:10.3389/fonc.2020.00966)
Supplement: Supplementary file 2 [file Table_2.DOCX]

**Supplement table 2.** Baseline Characteristics of GC patients in the Zhongshan Cohort (N=139).

| Clinical features | With peritoneal metastasis (n=59) | Without peritoneal metastasis  (n=80) | *P* value |
| --- | --- | --- | --- |
| Gender |  |  | 0.0002 |
| Male | 26 | 61 |  |
| Female | 33 | 19 |  |
| Age (median year, range) |  |  |  |
| Median | 57 | 62 |  |
| Range | 28-79 | 29-92 |  |
| Tumor localization |  |  | 0.0151 |
| Proximal | 4 | 19 |  |
| No proximal | 55 | 61 |  |
| Lauren type |  |  | 0.0001 |
| Intestinal type | 7 | 47 |  |
| Diffuse type | 38 | 12 |  |
| Mixed type | 14 | 21 |  |
| AEP expression |  |  | 0.0003 |
| Positive | 36 | 23 |  |
| Negative | 23 | 57 |  |
| Historical grade |  |  | 0.0002 |
| G1/G2 | 4 | 28 |  |
| G3/G4 | 55 | 52 |  |
| Her2 status |  |  | 0.1085 |
| Positive | 4 | 14 |  |
| Negative | 55 | 66 |  |
| Metastasis after radical surgery |  |  | 0.0735 |
| Yes | 27 | 50 |  |
| No | 32 | 30 |  |
| Palliative surgery |  |  | 0.6688 |
| Yes | 34 | 42 |  |
| No | 25 | 38 |  |
| Palliative chemotherapy |  |  | 0.1019 |
| Yes | 54 | 64 |  |
| No | 5 | 16 |  |
| Cycles of palliative chemotherapy |  |  | 0.7543 |
| < 4 cycles | 30 | 44 |  |
| ≥4 cycles | 29 | 36 |  |
| Median | 3 | 3 |  |
| Range | 0-10 | 0-9 |  |
| Metastasis |  |  |  |
| Liver | 3 | 61 |  |
| Lymph nodes | 13 | 20 |  |
| Ovary | 3 | 8 |  |
| Peritoneum | 59 | 0 |  |
| Lung | 0 | 6 |  |
| Bone | 2 | 6 |  |
| Anastomotic stoma | 0 | 6 |  |
